# Evidence of a tick RNAi pathway by comparative genomics and reverse genetics screen of targets with known loss-of-function phenotypes in *Drosophila*

**DOI:** 10.1186/1471-2199-10-26

**Published:** 2009-03-26

**Authors:** Sebastian Kurscheid, Ala E Lew-Tabor, Manuel Rodriguez Valle, Anthea G Bruyeres, Vivienne J Doogan, Ulrike G Munderloh, Felix D Guerrero, Roberto A Barrero, Matthew I Bellgard

**Affiliations:** 1 Cooperative Research Centre for Beef Genetic Technologies, Armidale, NSW, Australia; 2 Centre for Comparative Genomics (CCG), Murdoch University, Perth, Western Australia 6150, Australia; 3 Department of Primary Industries and Fisheries, Emerging Technologies, Locked Mail Bag No. 4, Moorooka 4105, Queensland, Australia; 4 Department of Entomology, University of Minnesota, St Paul, Minnesota 55108, USA; 5 USDA-ARS, Knipling Bushland US Livestock Insect Research Laboratory, 2700 Fredericksburg Road, Kerrville, TX 78028, USA

## Abstract

**Background:**

The Arthropods are a diverse group of organisms including Chelicerata (ticks, mites, spiders), Crustacea (crabs, shrimps), and Insecta (flies, mosquitoes, beetles, silkworm). The cattle tick, *Rhipicephalus (Boophilus) microplus*, is an economically significant ectoparasite of cattle affecting cattle industries world wide. With the availability of sequence reads from the first Chelicerate genome project (the *Ixodes scapularis *tick) and extensive *R. microplus *ESTs, we investigated evidence for putative RNAi proteins and studied RNA interference in tick cell cultures and adult female ticks targeting *Drosophila *homologues with known cell viability phenotype.

**Results:**

We screened 13,643 *R. microplus *ESTs and *I. scapularis *genome reads to identify RNAi related proteins in ticks. Our analysis identified 31 RNAi proteins including a putative tick Dicer, RISC associated (Ago-2 and FMRp), RNA dependent RNA polymerase (EGO-1) and 23 homologues implicated in dsRNA uptake and processing. We selected 10 *R. microplus *ESTs with >80% similarity to *D. melanogaster *proteins associated with cell viability for RNAi functional screens in both BME26 *R. microplus *embryonic cells and female ticks *in vivo*. Only genes associated with proteasomes had an effect on cell viability *in vitro*. *In vivo *RNAi showed that 9 genes had significant effects either causing lethality or impairing egg laying.

**Conclusion:**

We have identified key RNAi-related proteins in ticks and along with our loss-of-function studies support a functional RNAi pathway in *R. microplus*. Our preliminary studies indicate that tick RNAi pathways may differ from that of other Arthropods such as insects.

## Background

The understanding of gene function in a poorly studied Arthropod such as the cattle tick *Rhipicephalus (Boophilus) microplus *(subphylum Chelicerata: order Acari: suborder Ixodida) can benefit from the knowledge generated by genome-wide resources of the model insect *Drosophila melanogaster *(subphylum Mandibulata: order Hexapoda: suborder Insecta). The genome of the fruit fly *D. melanogaster *was among the first eukaryotic genomes to be sequenced and assembled [1]. *D. melanogaster *and *R. microplus *evolved from a common ancestor ca. 500 million years ago [2]. In comparison to the existing comprehensive genome resources for the fruit fly *D. melanogaster*, the cattle tick genome resources are limited to approximately 45,000 EST sequences [3]. In addition, the tick genome size of 7.1 Gbp [2] compared to the *D. melanogaster *of 139 Mbp [4] will likely delay the generation of a complete *R. microplus *genome sequence [5]. A genome project for the related tick species, *Ixodes scapularis*, with an estimated genome size of 2.1 Gbp, is currently underway [6]. Although there are many invertebrate genomes completed including worms, nematodes, beetle, wasp, honey bee, flies, and mosquitoes , *I. scapularis *will be the first Chelicerate:Arachnida genome sequence available representing mites, ticks, scorpions and spiders.

Among the many methods available for reverse genetic studies, RNA interference (RNAi) has gained popularity because of its demonstrated efficient post transcriptional gene-silencing effects in plants, fungi, nematodes, flies and cultured mammalians cells (reviewed by [7-11]). RNA mediated gene silencing is a widely conserved mechanism in eukaryotes and can be categorized into two partially overlapping pathways, the RNAi pathway and the microRNA (miRNA) pathway. The RNAi pathway is triggered by exogenous or endogenous dsRNAs that are recognized by Dicer RNase III proteins which 'dice' these molecules into double-stranded small interfering RNAs (siRNAs) of 21–23 nt in length [12]. A typical eukaryotic Dicer consists of 2 helicase domains, a PAZ domain, 2 RNAse domains and a dsRNA-binding domain (dsRBD) [12,13], however some variations in this domain structure have been noted for insect Dicers [14]. *D. melanogaster *has 2 Dicer enzymes, Dcr-1 and Dcr-2 which are responsible for miRNA and siRNA production respectively [15]. By contrast most other animals contain a single Dicer that generates both siRNAs and miRNAs.

The next phase in the RNAi pathway involves the loading of siRNAs into RNA-induced silencing complexes (RISCs). dsRNA binding motif proteins (dsRBM), such as *D. melanogaster *R2D2 and *Caenorhabditiselegans *Rde-4 help siRNAs to be loaded properly into silencing complexes [16,17]. Using the siRNAs as a guide, RISCs find target mRNAs and cleave them. Argonaute (Ago) family proteins are the main components of silencing complexes, mediating target recognition and silencing [18-20]. Most organisms have multiple members of the Ago proteins, for example both insect species *D. melanogaster *and *Tribolium castaneum *(beetle) have 5, whereas *C. elegans *(nematode) has 27 [14,21-25]. In *Drosophila *Ago-1 and Ago-2 are known to be associated with RISC [21]. In *C. elegans*, the primary siRNAs processed by Dicer can also trigger the amplification of siRNAs through a RNA-dependent RNA polymerase (RdRP) to produce secondary dsRNAs in a two-step mechanism involving secondary Argonaute proteins [26-28]. This mechanism has not been demonstrated in other animals to date and is commonly found in plants rather than animals.

An additional phenomenon identified in plants and *C. elegans *is the systemic spread of RNAi from cell to cell throughout the organism and its potential systemic transfer to subsequent generations through the germ-line [29-31]. Proteins related to this phenomenon in *C. elegans *include Sid-1, which encodes a multi-transmembrane domain protein thought to act as a channel for dsRNA uptake, and RNAi spreading defective proteins (Rsd-2, Rsd-3 and Rsd-6) shown to be required for the systemic RNAi response [32,33]. Originally, systemic RNAi was thought to be unique to *C. elegans *in animals, however preliminary evidence suggests that silkworm, honeybee, wasp and beetle utilize a Sid-1-like (sil) protein not found in mosquitoes or flies (reviewed by Tomoyasu et al [14]). Furthermore, over 20 genes identified as necessary for dsRNA uptake in *Drosophila *cultured cells have also been identified in other insect species [14,34-36]. The specific mechanisms associated with dsRNA uptake and systemic RNAi in Arthropods including some insect species are thus currently undefined.

Of the above described proteins associated with RNAi pathways, only one RNAi tick protein has been identified to date, a putative *R. microplus *Ago-2 [37]. RNAi pathways in Arthropods other than fruit flies and mosquitoes are beginning to demonstrate that there are evolutionary variations in these pathways with a higher level of divergence within the Arthropoda than previously thought [14,38-41]. Similarly, long dsRNAs have been successfully applied in *R. microplus *[42] and other tick species (e.g. *Amblyomma*, *Ixodes*, *Haemaphysalis*, and *Dermacentor *spp.) for targeted gene knockdown to demonstrate the function of tick-specific genes in various tick life stages, with some studies producing evidence of systemic RNAi spread into subsequent stages (reviewed by de la Fuente et al [37]). With the advent of increasing Arthropod genome resources, it may be feasible to identify more putative tick homologues of essential RNAi pathway-associated proteins to better elucidate the tick RNAi mechanism. Improving the understanding of the mechanisms used by ticks for gene knockdown will assist to develop specific tick RNA interference reagents and improved techniques for gene functional studies.

In this study we provide evidence for the presence of RNAi pathway associated proteins in *R. microplus *ESTs and *I. scapularis *genome reads, including a tick homologue for Dicer, Argonaute proteins, RdRP and proteins associated with dsRNA uptake and processing, and thus propose a putative tick RNAi pathway. We then determined whether targeting genes in the cattle tick which are homologous to *D. melanogaster *genes with known RNAi *in vitro *phenotypes [43] would similarly result in abnormal phenotypes. We identified 10 candidate genes and conducted *in vitro *and *in vivo *(female tick injections) RNAi loss-of-function assays. Interestingly, only proteasomal genes impaired tick cell viability *in vitro*, whilst 9 candidates impaired tick egg and larval development *in vivo*.

## Results

### Evidence of putative RNAi pathway in *R. microplus*

#### Dicer

A Dicer homologue was not confirmed in *R. microplus *however conserved domains commonly found in Dicer proteins of higher eukaryotes were identified in the *R. microplus *BmiGI2 EST database ([3](summarized in Additional File 1). A single *R. microplus *EST sequence (TC9337) was identified as containing an ORF of 250 amino acids (aa) encoding a putative RNase III (Pfam:PF00636) domain. The pairwise alignment of the ORF with the amino acid sequence of *C. elegans *Dcr-1 [GenBank:NP_498761] showed 24% identity and an e-value of 8e-12 (Additional File 1).

**Figure 1:**
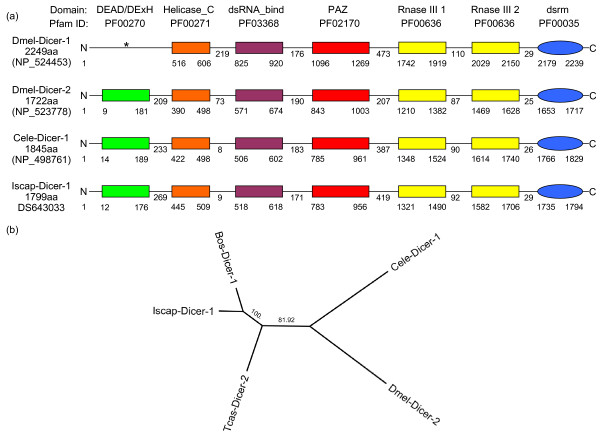
**The schematic domain structure of Dicer proteins**. (a) Comparison of the conserved domain structures of *D. melanogaster *Dicer-1 and Dicer-2, *C. elegans *DCR-1 and our predicted *I. scapularis *Dicer-1 protein. Names and IDs of the conserved domains are given as stored in the Pfam database. * = The Pfam search did not detect a signal for this domain in the sequence of the Dicer-1 protein of *D. melanogaster*. (b) Phylogenetic analysis of full-length Dicer proteins (Bos = *B. taurus*, Cele = *C. elegans*, Dmel = *D. melanogaster*, Iscap = *I. scapularis*, Tcas = *T. castaneum*).

A Dicer tick homologue with the expected domain structure for a eukaryotic Dicer was identified in *I. scapularis *in a recently assembled supercontig [GenBank: DS643033] from the Ixodes Genome Project (IGP) [6]. This supercontig represents a 350 kb region of the *I. scapularis *tick genome and we identified a 22.3 kb genomic region containing a single gene that has 14 exons (the annotation described here has been submitted to the IGP). The predicted *I. scapularis *Dicer protein is 1799aa long and has 31% similarity to the predicted Dicer-1 isoform 4 from the dog *Canis lupus familiaris *[GenBank:XP_868526]. Furthermore, Figure 1a shows that the predicted *I. scapularis *Dcr-1 homologue has the same domain composition as its counterparts in *D. melanogaster *and *C. elegans*. The identified *I. scapularis *Dicer homologue clusters with the Dicer protein from the bovine *Bos taurus *(Figure 1b).

#### Argonaute proteins

**Figure 2:**
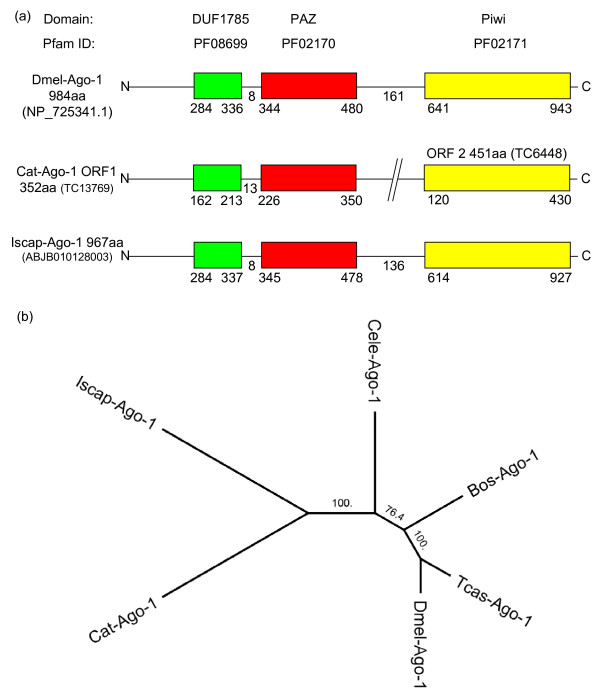
**Domain structure and phylogenetic tree of tick Argonaute-1 proteins**. (a) Schematic structure of the Argonaute-1 proteins from *D. melanogaster *and our predictions of the structures of the *I. scapularis *and *R. microplus *Argonaute-1 homologues. (b) Phylogenetic analysis of Argonaute-1 proteins. (Bos = *B. taurus*, Cat = *R. microplus*, Cele = *C. elegans*, Dmel = *D. melanogaster*, Iscap = *I. scapularis*, Tcas = *T. castaneum*).

**Figure 3:**
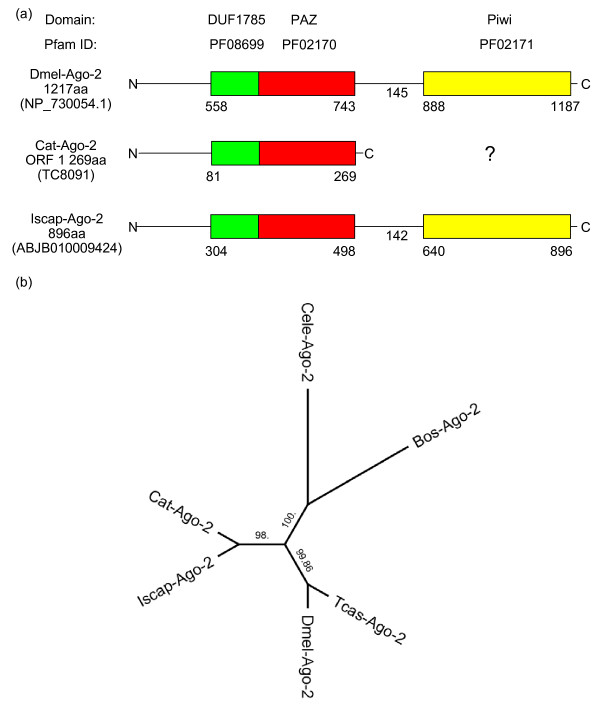
**Domain structure and phylogenetic tree of tick Argonaute-2 proteins.** (a) The structure of the predicted Argonaute-2 proteins from *I. scapularis *and *R. microplus *in comparison to the structure of Argonaute-2 in *D. melanogaster*. The predicted structural property of both tick Argonaute-2 candidates is similar to the structure of the fruit fly Argonaute-2 protein. No *R. microplus *ORF with a Piwi domain similar to Argonaute-2 Piwi was identified. (b) Phylogenetic analysis of Argonaute-2 proteins (Bos = *B. taurus*, Cat = *R. microplus*, Cele = *C. elegans*, Dmel = *D. melanogaster*, Iscap = *I. scapularis*, Tcas = *T. castaneum*).

The analysis of *R. microplus *ESTs and *I. scapularis *genome reads identified putative tick homologues of *D. melanogaster *Argonaute-1 and 2 proteins in both species (Table 1, Figures 2 and 3).

**Table 1:** Putative tick RNAi candidate homologues

**Function**	*Protein	***R. microplus *BmGI2 ID (% Identity)^±^**	***I. scapularis *contig ID (% Identity)**	**Ref**
**DICER **(See also Figure 1 & Additional File 1)				
RNase III dsRNA processing	*Dcr-1^Cele^	incomplete	DS643033 (31%)	[12,13]
**Argonaute proteins – target recognition and silencing **(See also Figures 2 & 3)				
RISC – miRNA pathway	Ago-1	TC13769 (43% DUF1785 and PAZ domains), TC6448 (44% PIWI domain)	ABJB010009424.1 (41%)	[21]
RISC – RNAi pathway	Ago-2	TC8091 (25% DUF1785 & PAZ domains)	ABJB010128003.1 (31%)	[18]
**Systemic RNAi (germ cells)**	*Rsd-3^Cele^	MPAAN09TR (45%)	ABJB010279725.1 (48%)	[32]
**dsRNA uptake and processing**				
Endocytic protein (EPsiN)	*Epn-1^Cele^	BEADR88TR (71%)	ABJB010748067.1 (43%)	[32]
Vesicle mediated transport	AP-50	TC6127 (89%)	ABJB010508398.1 (91%)	[35]
	Arf72	Not found	ABJB010115816.1 (68%)	[35]
	Chc (Clathrin hc)	TC10346 (60%	ABJB010065986.1 (87%)	[35]
Endosome transport	Rab7	BEAGW52TR (80%)	ABJB010159881.1 86%)	[35]
Intracellular transport	CG3911	TC6954 (67%)	ABJB010384785 (64%)	[35]
	Cog3	TC5984 (49%)	ABJB010296208.1 (68%)	[35]
	ldlCp	Not found	ABJB011123114.1 (52%)	[35]
Lysosomal transport	Lt	TC12854 (35%)	Not found	[35]
Lipid metabolism	Gmer	TC9381 (62%)	Not found	[35]
	Pi3K59F	BEADT89TR (52%)	Not found	[35]
	Sap-r	TC9046 (22%)	Not found	[35]
Proteolysis and peptidolysis	CG4572	TC6395 (35%)	ABJB010180836.1 (43%)	[35]
	CG5053	Not found	ABJB010804049.1 (63%)	[35]
	CG8184	Not found	ABJB010385401.1 (72%)	[35]
Oogenesis	Egh	TC8075 (67%)	ABJB010259843.1 (66%)	[35]
Rhodopsin mediated signaling	ninaC	Not found	ABJB011087029.1 (42%)	[35]
Translation regulation	Srp72	Not found	ABJB010441811.1 (38%^)^	[35]
ATP synthase/ATPase	Vha16	MPAA174TR (59%)	ABJB010975295.1 (67%)	[35]
	VhaSFD	TC10823 (63%)	ABJB010753004 (56%)	[35]
Unknown	CG5161	TC14816 (61%)	No found	[35]
	CG5382	Not found	ABJB010478954.1 (84%)	[35]
**Other factors**				
RISC assembly	Armitage	TC9347 (35%)	Not found	[73]
RISC associated nuclease	TudorSN	BEAFW62TR (46%)	ABJB010481234.1 (48%)	[46]
RISC function	FMRp	BEAE145TR (53%)	ABJB010028120.1 (67%)	[50]
ATP-dependent RNA helicase	Rm62	TC14966 (70%)	ABJB010043214.1 (54%)	[45]
RNA-directed RNA polymerase (see also Figure 4)	*EGO-1^Cele^	BEAEL55TR (41%)	ABJB010057970.1 (54%)	[28]

*All proteins of the RNAi pathway originate from *D. melanogaster*, except:^Cele ^= *C. elegans; *Not found homologues include: *C. elegans *Rsd-2, Rsd-6, Sid-1, Tag-130 (systemic RNAi), Rde-1, Rde-4 (associated with RNAi machinery); *T. casteneum *Tc-Sil (systemic RNAi); *D. melanogaster *Eater, Sr-CI, Sr-CII, Sr-CIII, Sr-CIV (Innate immune response/phagocytosis – dsRNA uptake and processing), CG5434, CG8671 (Unknowns – dsRNA uptake and processing), R2D2, Vasa intronic gene, and Spindle E (associated with RNAi machinery).

^± ^GenBank accessions for *R. microplus *tentative consensus sequences and clones are listed in Additional file 6.

Figure 2a summarizes the domain structure of the identified tick Ago-1 proteins. The cattle tick Argonaute-1 protein (Cat-Ago-1) is partially encoded by two ESTs (Figure 2a). TC13769 encodes an ORF of 352aa containing the DUF1785 domain located from aa 162 to 213 and a PAZ domain from aa 226 to 350. A pairwise alignment using blastp showed 43% identity with the respective domains of *D. melanogaster *Argonaute-1 protein. Another EST, TC6448 encodes the putative Piwi domain of the Cat-Ago-1 protein, which has 46% identity with the Piwi domain of *D. melanogaster *Ago-1. The Piwi domain encoded in TC6448 is located from aa 120 to 430. The *I. scapularis *contig ABJB010128003.1 (Iscap-Ago-1) encodes an ORF of 967aa containing all three known domains of Ago-1 proteins (Figure 2a). The PAZ domain of Iscap-Ago-1 has 41% and 47% similarity with the PAZ domains of Dmel-Ago-1 and Cat-Ago-1, respectively. Interestingly, the Piwi domain of Iscap-Ago-1 shows a higher sequence similarity, being 52% and 57% identical to its counterpart in *Drosophila *and cattle tick. Based on the multiple sequence alignments of the DUF1785 and PAZ of the Argonaute-1 proteins a clustering of *I. scapularis *and *R. microplus *is observed (Figure 2b). The second cluster consists of the sequences from the insect species *T. castaneum *and *D. melanogaster*. The sequences of the Argonaute-1 proteins from *C. elegans *and *B. taurus *form two separate outlying groups.

Figure 3a summarizes the domain structure of the identified tick Ago-2 proteins. TC8091 represents a putative cattle tick Argonaute-2 protein (Cat-Ago-2) encoding an ORF of 269aa harboring the DUF1785 and PAZ domains located from aa 81 to 134 and aa 135 to 269 (27% identity), respectively. The *R. microplus *Ago-2 homologue TC984 (TC9244/TC16832, BmiGI2) identified by de la Fuente et al [37] was also confirmed in our search and appears to encode a Piwi domain. Interestingly, the pairwise alignment using blastp with both *D. melanogaster *Argonaute proteins showed an overall identity of 42% with Argonaute-1 and 39% with Argonaute-2. The *I. scapularis *contig ABJB010009424.1 (Iscap-Ago-2, Figure 3a) encodes an ORF of 896aa consisting of a DUF1785 domain from aa 304 to 357, a PAZ domain from aa 358 to 498 and a Piwi domain located in the region from aa 640 to 896. Sequence comparison of the putative Iscap-Ago-2 with the Dmel-Ago-1 ([GenBank:NP_725341.1], 27% identity) and Dmel-Ago-2 ([GenBank:NP_730054.1], 41% identity) proteins revealed a higher homology between Iscap-Ago-2 and Dmel-Ago-1, nevertheless the predicted domain structure of this putative *I. scapularis *Argonaute was more similar to Dmel-Ago-2 (Figure 3a). The phylogenetic tree of the Argonaute-2 proteins shows three clusters (Figure 3b). The *I. scapularis *and *R. microplus *sequences group together, the second cluster consists of the Argonaute-2 proteins from *D. melanogaster *and *T. castaneum*, and in the third group the sequences from *C. elegans *and *B. taurus *are clustered.

#### Systemic RNAi and dsRNA uptake/processing

Available *R. microplus *ESTs and *I. scapularis *genomic contigs were screened for homologous genes to *C. elegans *proteins involved in RNAi systemic spread (Rsd-2, Rsd-3, and Rsd-6) and dsRNA uptake (Epn-1) (Table 1). We identified putative hits to Rsd-3 and Epn-1 in both *R. microplus *and *I. scapularis *with 45% and 48% (Rsd-3) and 71% and 43% (Epn-1) identities, respectively. Screening for tick homologues against the 30 *D. melanogaster *proteins implicated in dsRNA uptake and processing [35] identified 14 and 16 homologues in *R. microplus *and *I. scapularis*, respectively, at varying levels of similarity for the 23 hits (22–91%). The highest similarity was observed with dsRNA uptake homologues associated with vesicle mediated transport, intracellular transport, oogenesis, endosome transport and ATPase for both tick species (Table 1).

#### RISC components and RdRP

**Figure 4:**
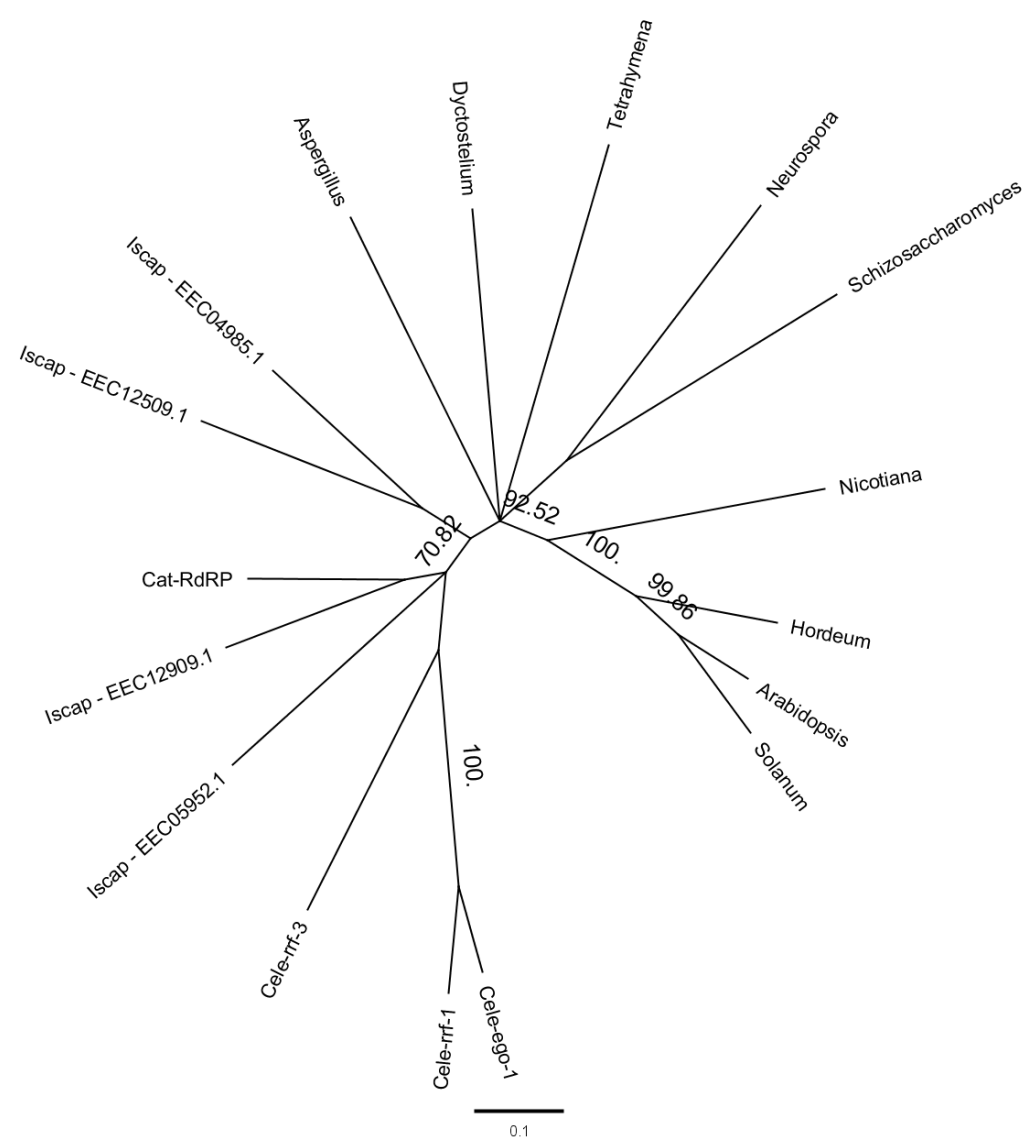
**Phylogenetic tree constructed from the multiple sequence alignment of the partial *R. microplus *RdRP domain (Cat-RdRP) and hypothetical *I. scapularis *RdRP proteins (Iscap) to RdRP sequences from selected plants (*Nicotiana tabacum*, *Hordeum vulgare*, *Arabidopsis thaliana*, *Solanum lycopersicum*), fungi (*Schizosaccharomyces pombe*, *Neurospora crassa *and *Aspergillus fumigatus*), protists (*Tetrahymena thermophila *and *Dictyostelium discoideum*) and the metazoan *C. elegans *(Cele-ego-1, Cele-rrf-1/3)**. The branch labels display the consensus support in %.

Similarity searches with the protein sequences of the putative RNA helicases Armitage and Rm62, involved in the assembly of RISC, resulted in best hits on *R. microplus *sequences TC9347 and TC14966 respectively but no homologues of Spindle E were found (Table 1). Searches using *D. melanogaster *protein sequences for FMRp and TudorSN returned best hits on the *R. microplus *ESTs BEAE145TR (53% identity) and BEAFW62TR (46% identity), respectively. It must be noted that only one SN domain was identified in the putative homologue which either indicates that it is not a true TudorSN homologue or that the consensus sequence is currently incomplete. However, a recent GenBank submission indicates the presence of a putative *I. scapularis *TudorSN identified simultaneously with this study ([GenBank:EEC18716.1] *Ixodes scapularis *Genome Project Consortium). The EGO-1 protein from *C. elegans *has RNA-directed RNA polymerase (RdRP) activity and is associated with the *C. elegans *transitive RNAi pathway by amplifying the trigger dsRNA and/or siRNAs [28]. The EST BEAEL55TR exhibited a 41% identity with the *C. elegans *RdRP – EGO-1 (Table 1). Nine putative *I. scapularis *RdRP accessions have been deposited into GenBank by the *Ixodes scapularis *Genome Project Consortium simultaneously with this study. A total of 4 of these *I. scapularis *RdRP sequences share conserved regions with the partial *R. microplus *RdRP and thus the new Accessions EEC04985.1, EEC05952.1, EEC12509.1 and EEC12909.1 were utilized for the *I. scapularis *RdRP sequences in the consensus tree presented in Figure 4. All 5 tick RdRPs demonstrate a close phylogenetic relationship with the partial *R. microplus *RdRP clustering with *I. scapularis *EEC12909.1. *I. scapularis *sequences EEC12509.1 and EEC04985.1, and EEC05952.1, form separate branches respectively. The RdRP proteins from *C. elegans *form another distinct cluster with the tick RdRPs branching between *C. elegans *and those from fungi, plants and protists (Figure 4). Apart from the Armitage homologue, all *R. microplus *hits associated with RISC components and RdRP above were confirmed in *I. scapularis *genome reads in this study (Table 1).

#### Summary of putative tick RNAi pathway

**Figure 5:**
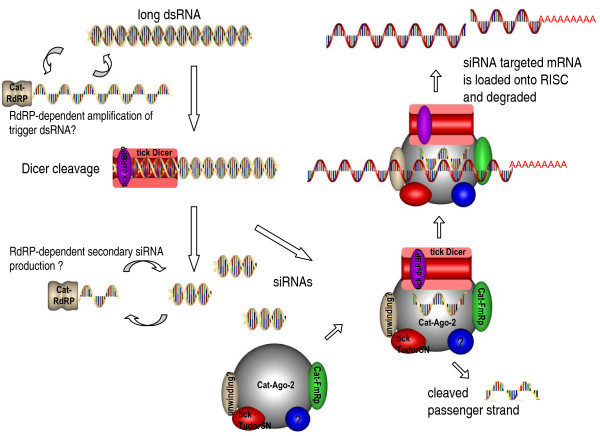
**Schematic representation of a putative tick RNAi pathway**. Cattle tick homologues are indicated using a 'Cat' prefix for proteins where *Rhicipephalus *(*Boophilus*) *microplus *homologues are identified in this study (GenBank Accessions are listed in Additional File 6). The proposed activity of the Cat-RdRP (RNA dependent RNA polymerase, EGO1-like) is indicated as amplifying trigger dsRNA or cleaved siRNAs. Long dsRNAs are recruited to Dicer (putative tick Dicer identified in *I. scapularis *genome reads) via a yet to be identified dsRNA Binding Protein. The RNA-Induced-Silencing Complex (RISC) includes a Cat-Ago-2 (Argonaute-2 homologue), tick TudorSN (*I. scapularis *tudor-staphylococcal nuclease – GenBank EEC18716.1) and a Cat-FmRp (representing the *D. melanogaster *orthologue of the fragile-X mental-retardation protein essential to RISC). Homologues for a tick RNA unwinding protein and a vasa intronic gene (associated with RISC) were not identified. The schematic diagram was partly adapted from Sontheimer 2005 [44] and was drawn using Solid Edge Version 20 (Siemens PLM Software, TX, USA).

Figure 5 shows a schematic diagram of a putative tick RNAi pathway. Putative proteins identified in *R. microplus *ESTs have been described using the 'Cat' (Cattle tick) prefix. dsRNA is taken up by tick cells and the RNAi effect spreads to subsequent tick stages by an unknown mechanism [42]. It is yet unconfirmed whether a SID-1 or Sil-1 homologue exist in ticks, however, it is feasible that RdRP and associated proteins are involved in germ-line spread similar to the *C. elegans *RdRP pathway [28]. Here we postulate the potential amplification of both trigger dsRNA and secondary siRNAs through the involvement of a Cat-RdRP. A *R. microplus *Dicer was not identified, although a homologue was identified in the *I. scapularis *genome reads as described above. A definitive dsRNA binding protein (such as *D. melanogaster *R2D2 or *C. elegans *Rde-4) potentially associated with Dicer was not found using the current tick sequence resources. A confirmed tick Rde-1 was not identified but has been associated with the RdRP pathway if present [25]. Dicer guides the siRNA to the RISC structure which has been adapted from the Sontheimer RNAi published diagram [44]. The RISC structure demonstrates homologues for a cattle tick *Drosophila *Fragile × protein (Cat-FMRp), tick TudorSN (*Ixodes scapularis *Genome Project Consortium) and the Cat-Ago-2 described above [44-46]. Other proteins putatively associated with dsRNA uptake, systemic or germ-line RNAi listed in Table 1 were not included in this diagram.

### Selection of *R. microplus *conserved homologues for RNAi gene silencing

To validate further a putative functional RNAi pathway in *R. microplus *we conducted RNAi-mediated loss-of-function assays *in vitro *and *in vivo*. We first selected tick RNAi targets based on their homology to *Drosophila *genes known to display an RNAi phenotype [43]. Of the 438 *Drosophila *genes known to affect growth and viability, 40 were identified in the *I. scapularis *genome reads and 37 in the *R. microplus *BmiGI2 database with 31 hits common between the tick species (results not shown). These results were based on blastn searches with an e-value cut-off of <1e-10. To select the most conserved sequences for tick *in vitro *studies, using high stringency searches (>80% identity, e-value <1e-50), 11 *R. microplus *ESTs were identified in the BmiGI2 database as homologous to *D. melanogaster *genes with RNAi phenotypes affecting growth and viability at z scores >3 [43] (Table 2). An additional 2 highly conserved homologues were selected as negative controls, one with a lower z score (*Drosophila string of perls*) and one with a nil z score thus with a nil effect on cell culture growth and viability (*Drosophila Tat-binding protein-1*), Table 2. The putative function of these 13 *R. microplus *ESTs were then assigned by retrieving the annotated InterPro domains of their *Drosophila *counterparts (Table 2). Evaluation of the assigned functional information revealed that 5 sequences putatively have a role in ribosome and protein synthesis (TC5762, TC9037, TC12306, TC12372, TC12393), 4 in proteasome and ubiquitinylation (TC6372, TC9852, TC10417, TC13930), 3 in DNA binding (TC6116, TC12182, TC9417), and one in energy and metabolism (TC5823). The controls used were the *Drosophila string of perls *(TC5762) and *Tat-binding protein-1 *(TC13930) homologues respectively.

**Table 2:** *R. microplus *homologues with high conservation (≥ 80% identity) to 13 *D. melanogaster *proteins following RNAi knockdown *in vitro *(11 associated with significant cell viability z scores at >3 and 2 controls at<3)

*R. microplus *BmiGI2 Reference*	*D. melanogaster *description			*D. melanogaster *cell culture RNAi cell growth and viability Z-scores^§^	
	Functional group assignment	Gene Symbol (Name)	InterPro Domains ID (Name)	Kc167	S2R+
TC5762	Ribosome and protein synthesis	sop (*string of perls*)	IPR000851 (Ribosomal Protein S5)	2.9^¶^	2.9^¶^

TC5823	Energy and metabolism	ATPsyn-(beta) (*ATP synthase-(beta)*)	IPR000194 (ATPase, F1/V1/A1 complex, alpha/beta subunit, nucleotide binding)	5.0	2.0

TC6116	DNA binding	His3.3A (*Histone H3.3A*)	IPR000164 (Histone H3)	2.4	3.9

TC6372	Proteasome and ubiquitin	Ubi-p63E (*Ubiquitin-63E*)	IPR000626 (Ubiquitin)	7.7	5.9

TC9037	Ribosome and protein synthesis	RpL11-PA (*Ribosomal protein L11*)	IPR002132 (Ribosomal Protein S5)	3.2	3.0

TC9417	DNA binding^±^	CG2807	IPR000357 (Heat)	4.3	6.1

TC9852	Proteasome and ubiquitin	Pros26.4 (*Proteasome 26S subunit subunit 4 ATPase*)	IPR003593 (AAA ATPase)	4.8^§^	2.8

TC10417	Proteasome and ubiquitin	Rpt1	IPR003593 (AAA ATPase)	4.5^§^	2.9

TC12182	DNA binding	His3.3A (*Histone H3.3A*)	IPR000164 (Histone H3)	2.4	3.9

TC12306	Ribosome and protein synthesis	RpL8 (*Ribosomal protein L8*)	IPR002171 (Ribosomal Protein L2)	3.2	3.4

TC12372	Ribosome and protein synthesis	RpL10Ab (*Ribosomal protein L10Ab*)	IPR002143 (Ribosomal Protein L1)	2.3	3.7

TC12393	Ribosome and protein synthesis	RpS13 (*Ribosomal protein S13*)	IPR000589 (Ribosomal Protein S15)	3.4^§^	1.0

TC13930	Proteasome and ubiquitin	Tbp-1 (*Tat-binding protein-1*)	IPR003593 (AAA ATPase)	0^¶^	0^¶^

*GenBank accessions for *R. microplus *tentative consensus sequences and clones are listed in Additional file 6

^± ^Unassigned in Boutros et al. (2004) [43]

^§ ^Lethal *in vivo *(dsRNA injected into embryo) (flybase)

^¶ ^Tbp-1 was included as culture control – no viability effects following dsRNA knockdown in *Drosophila *culture [43]; sop (string of perls) knockdown *in vitro *was not considered significant in the *Drosophila *study. Both targets were included in this study as putative negative controls

Although all primers for target amplification prior to RNA transcription were designed by targeting conserved consensus regions as described in the methods, amplification of TC5823 (energy and metabolism/ATP biosynthesis), TC9417 (DNA binding) and TC12372 (ribosome and protein synthesis) was inconsistent with poor yields which were inadequate for RNA transcription (not shown). dsRNAs were transcribed successfully for the remaining 10 target genes (8 high z scores, 2 controls) used for RNAi experiments in cultured *R. microplus *BME26 cells and adult female ticks.

### Gene silencing in cultured tick cells

**Figure 6:**
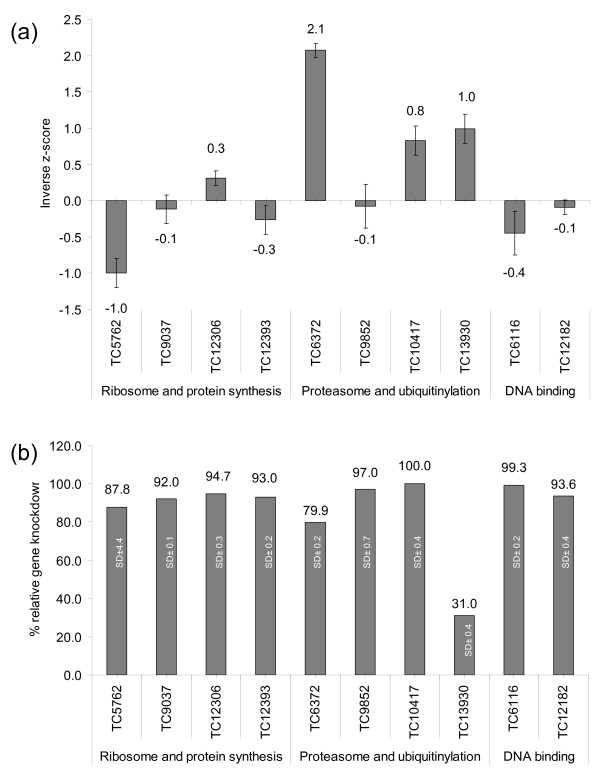
**Cell culture knockdown**. (a). Growth and viability RNAi phenotypes expressed as inverse z-scores of genes involved in ribosome and protein synthesis (TC5762, TC9037, TC12306, TC12393), encoding proteasome components and participating in ubiquitinylation (TC6372, TC9852, TC10417, TC13930), and having DNA binding functions (TC6116, TC12182). A positive z-score indicates reduced cell growth and viability. (b). Effect of dsRNA-induced knockdown on RNAi targets measured by quantitative RT-PCR and presented as % of gene expression levels relative to the housekeeping gene.

None of the dsRNA treatments had significant effects on tick cell viability (Figure 6a) compared to controls and compared to z scores >3 as described for the same targets in *Drosophila *cells (Table 2, [43]). However, TC6372 (*Ubiquitin-63E *homologue) knockdown demonstrated the most severe effect on growth and viability (inverse z score 2.1) (Figure 6), also confirmed by microscopic examination of the cells (not shown). An additional 2 target genes (*Rpt1 *TC10417, and *Tat-binding protein-1 *TC13930) demonstrated a slight reduction on cell viability with z scores 0.8 and 1.0 respectively. It is feasible that the TC13930 treatment may have had a stronger viability phenotype if the knockdown had been more effective (only 31% compared to other treatments ~>79%). Collectively the effects are less significant in tick cells than for their *Drosophila *counterparts in *Drosophila *cells, these 3 treatments are all associated with proteins involved in proteasome and ubiquitin function. Quantitative RT-PCR analysis confirmed that all RNAi targeted genes resulted in a substantial reduction of the corresponding target mRNA (79.9 – 100%) except for TC13930 at 31% (Figure 6b).

### Gene silencing in adult female ticks (reproduction phenotype)

**Figure 7:**
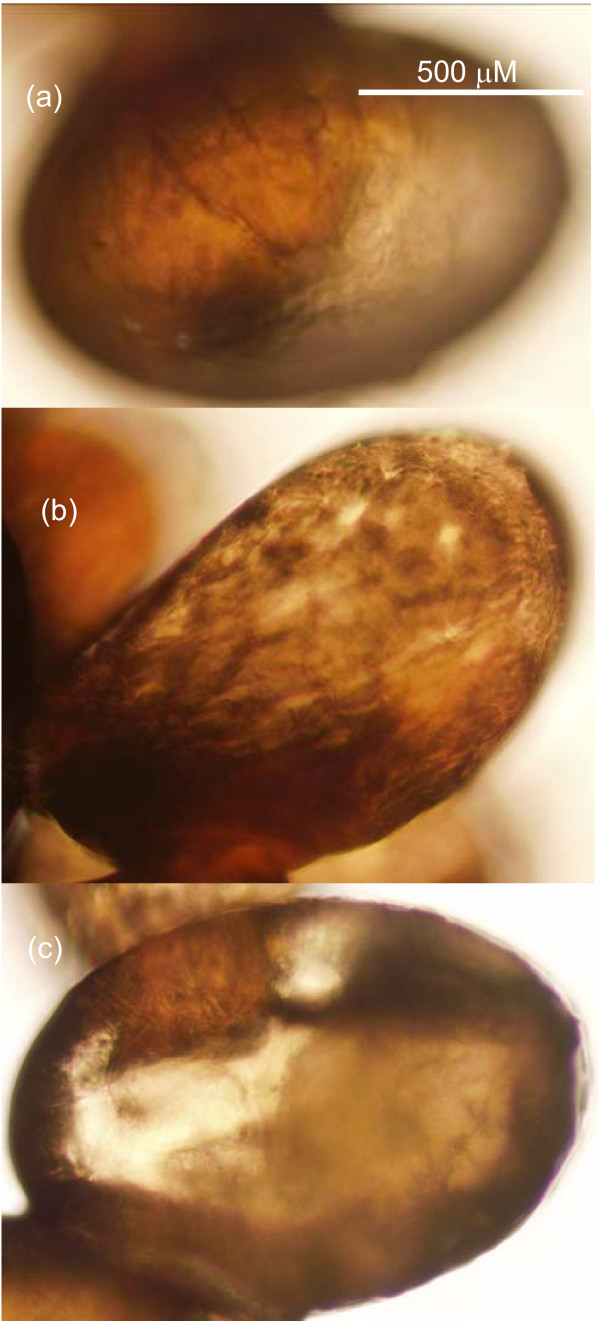
**Differences in egg morphologies following treatment of *R. microplus *adult female ticks with dsRNA**. (a) Egg from untreated females approximately 15 day after laying, (b) Eggs from females treated with TC6372 (*D. melanogaster Ubiquitin 63E-like *transcript) dsRNA approximately 15 days after laying. (c) Eggs from females treated with dsRNA targeting TC12306 (*D. melanogaster ribosomal protein L8-like *transcript) approximately 15 days after egg laying.

The same treatments were tested in live adult female ticks to measure any *in vivo *effects of gene silencing on tick survivability, egg output and larval hatching. Eggs laid by ticks from the control groups showed no obvious morphological changes (see Figure 7a for eggs from "no treatment" group). The average egg mass weight was 0.118 g for the control dsRNA group, 0.134 for the tick actin dsRNA group, 0.107 g for the PBS injection control group and 0.128 g for the negative control group (nil injection). Eggs from the control groups showed a normal embryonic development time to larval hatching at 27 days. The larval hatching rates for control treatments ranged between 62.0–69.8% (Table 3).

**Table 3:** Effect of tick *in vivo *dsRNA gene knockdown treatments on female tick survival and subsequent reproduction fecundity by targeting *Drosophila *homologues described in Table 2

**dsRNA treatment**			**Average (5 replicates per treatment)**					**qRT-PCR % knockdown (average 3 replicate reactions)**	
**Treatment**	***R. microplus *target BmiGI2 ID**	***D. mel *homologue**	**Tick survival (days)**	**Egg output (g)**	**Egg morphology (see Figure 6)**	**Days from laying to larval hatch**	**% larval hatch**	**Viscera (adult ticks)**	**Eggs **

dsRNA control (MEGAScript)	NA*	NA*	16.4	0.118	normal	26.9	69.8		
PBS injection	NA*	NA*	15.0	0.107	normal	26.5	62.0		
No injection	NA*	NA*	14.8	0.128	normal	27.1	64.9		
Actin	TC12168	NA*	16.2	0.134	normal	26.3	65.8		
Proteasome and ubiquitin	TC6372	*Ubiquitin-63E*	10.0	0.010	deformed	NL^±^	0.0	95.6	ND^§^
	TC9852	*Proteasome 26S subunit ATPase*	17.0	0.116	deformed	NL^±^	0.0	98.0	76.0
	TC10417	*Rpt 1*	16.4	0.130	normal	26.2	65.1	94.0	ND^§^
	TC13930	*Tat-binding protein-1*^¶^	16.4	0.127	slow development	32.9	0.4	99.0	76.0
Ribosome and protein synthesis	TC5762	*string of perls*^¶^	17.6	0.129	deformed	NL^±^	0.0	99.9	100.0
	TC9037	*Ribosomal Protein L11*	14.4	0.110	deformed	NL	0.0	21.3	98.9
	TC12306	*Ribosomal protein L8*	17.0	0.094	deformed	NL^±^	0.0	99.2	ND^§^
	TC12393	*Ribosomal protein S13*	16.6	0.135	deformed	NL^±^	0.0	99.0	ND^§^
DNA binding	TC6116	*Histone H3.3A*	15.8	0.114	slow development	30.7	3.1	97.6	100.0
	TC12182	*Histone H3.3A*	16.4	0.121	normal	27.8	35.2	99.2	94.7

^#^LSD (P = 0.05)			2.1	0.029		1.3	17.7		

*NA (not applicable) – control injections (no dsRNA treatment and *R. microplus *actin control)

^± ^NL (no larvae) indicates no larvae hatched

^§ ^ND (not done) indicates insufficient total RNA to undertake RT-PCR due to poor egg output.

^¶ ^Tat-binding protein 1 had a nil z score in the *D. melanogaster *study and string of perls had a z score <3 [43]

^#^LSD = least significant difference values

*Ubiquitin-63E *dsRNA treatment had the most significant effect on adult tick survival (average 10 days, approximately 5 days less than the controls, Table 3). Eggs laid by *R. microplus *females injected with dsRNA targeting genes associated with ribosome/protein synthesis (TC5762/*string of perls*; TC12306/*Rpl-8*; TC9037/*Ribosomal protein L11*; TC9852/*Proteasome 26S subunit*; TC12393/*Ribosomal protein S13*) and proteasome/ubiquitin (TC6372/*Ubiquitin-63E*) demonstrated the most lethal effect on tick reproduction with deformed egg morphology and no larvae hatching (Table 3). RNAi targeting of TC12306 (*Rpl-8*) and TC6372 (*Ubiquitin-63E*) generated the greatest reductions in average egg output, with *Ubiquitin-63E *(TC6372) treated ticks again being significantly affected compared with the controls (Table 3). Figures 7b and 7c show examples of phenotypic effects on embryo development due to TC6372 (dehydrated in appearance, embryo not visible) and TC12306 (embryo smaller in size) knockdown respectively. Down-regulation of the TC13930/*Tat-binding protein-1 *also associated with proteasome/ubiquitin function, impaired embryo development leading to a poor larval hatching rate (0.4%). In contrast, another gene associated with proteasome/ubiquitin (TC10417/*rpt 1*) had no effect on egg development and hatching rates compared with controls, although this target demonstrated a decrease in cell viability in cell culture experiments above. The 2 ESTs associated with DNA binding (TC6116 and TC12182/*histone H3.3A*) both induced slower embryo development and reductions in egg hatching at 3.1% and 35.2% respectively. A single (sixth) tick was harvested to confirm the relative reduction of transcript levels (% knockdown determined by qRT-PCR) for both the adult ticks and eggs (where applicable) for each *Drosophila *homologue treatment. All treatments except TC9037 demonstrated high knockdown of transcripts in adult tick viscera (≥ 94%), with knockdown also confirmed in eggs tested (≥ 76%). As the viscera from only one tick per treatment was harvested for RT-qPCR, it is feasible that the TC9037 treatment on this single tick was not delivered successfully, however, none of the 5 ticks injected produced viable eggs indicating a knockdown phenotype for the TC9037 treatment overall.

Thus 9 of the 10 *Drosophila *tick homologues demonstrated either a lethal effect on reproduction or a reduction in larval hatching rates when using dsRNA targeted knockdown *in vivo*. The significant effects associated with the *Ubiquitin-63E *homologue TC6372 treatments correlates with the highest z scores for the same target in *Drosophila *cell viability out of the sub-set of targets used here (Table 2, [43]). Data from the FlyBase website  identified that *in vivo *studies involving dsRNA injection into *Drosophila *embryos was lethal for both *Rpt1 *and *RpS13 *(correlating to TC10417 and TC12393 above). However, *Rpt1 *(TC10417) was the only treatment which did not have an effect *in vivo *for ticks. Fly *in vivo *knockdown studies associated with the 8 of the remaining targets were not found.

## Discussion

The lack of tick genome sequence resources has limited the ability to mine for RNAi protein homologues however research to date has suggested that ticks utilize a dsRNA-mediated RNAi similar to that described in insects such as flies and mosquitoes [47,48]. De la Fuente and colleagues [37] postulated a model for tick dsRNA-mediated RNAi following the identification of a putative Ago-2 protein in the *R. microplus *EST database. Our results support the diagram represented in de la Fuente et al [37] demonstrating evidence for putative tick Dicer, RISC associated proteins and dsRNA uptake homologues, however we identified a *R. microplus *EGO-1 homologue known to be implicated in RNA-directed RNA polymerase activity previously not identified in animals other than *C. elegans*. We also identified a Cat-Ago-2 at higher similarity than the tick homologue identified by de la Fuente et al [37] which exhibited higher similarity to the Argonaute-1 protein of *D. melanogaster *in this study. This is the first comprehensive analysis of RNAi sequence domains for a tick species and for the Chelicerate Arthropods.

Compared to the vast insect genome resources (flies, mosquitoes, beetle, silkworm, wasp – to name a few), there is currently only one Chelicerate genome available with the *I. scapularis *tick genome project nearing completion. To provide an evolutionary perspective to demonstrate relationships within the Ecdysozoan infraphylum it is important to note that their common ancestors may have existed over 1 billion years ago [49]. Comparative genomics between these phyla is in its infancy and pathways such as RNAi interference and gene regulation to date have been based on the fruit fly *D. melanogaster *as the model organism. As differences between RNAi pathway mechanisms between *C. elegans *and *D. melanogaster *are evident, it is thus feasible that Chelicerates could also vary from insects albeit their evolutionary distance is less (~500 million years) [2]. Indeed, definitive hits for the domains and proteins here were not exclusive to the Arthropoda, with the putative tick RNAi proteins matching homologues in diverse species such as insects (beetle, silkworm, wasp), nematodes, and mammals (data not shown).

While only a single Dicer protein is present in mammals and in *C. elegans*, in *D. melanogaster *siRNAs and miRNAs are produced by distinct Dicer enzymes [15]. In this study we identified one putative tick Dicer in the *I. scapularis *genome reads however evidence for more than one Dicer cannot at this stage be confirmed. This *I. scapularis *Dicer was found to be most similar to a Dicer-1 from a mammal. A *R. microplus *Dicer could not be confirmed, however an *I. scapularis *homologue was also not previously identified using EST data alone. Our preliminary evidence points to a 'single' tick Dicer with yet un-confirmed structure, though until complete tick genome resources are available the presence of more than one Dicer cannot be entirely dismissed.

The RISC structure contains the following essential proteins: *D. melanogaster *R2D2 or *C. elegans *Rde-4 [17], *D. melanogaster *homologue of the Fragile × mental retardation protein (FMRP) dFXR [45], Vasa Intronic gene (VIG) and a Tudor Staphylococcal nuclease [46,50]. We were able to confirm the presence of putative tick FMRp but no significant hits for TudorSN or VIG homologues and no significant similarity to known RNA binding proteins using the current tick resources. However, a concurrent study has identified a putative *I. scapularis *TudorSN (GenBank, *Ixodes scapularis *Genome Project Consortium) yet to be confirmed in *R. microplus*. The Argonaute (Ago) family of proteins contains 2 distinct RNA-binding domains PAZ and PIWI (PPD) required to bind the siRNA and to slice the cognate RNA to be degraded, respectively and thus are essential to RISC [51,52]. Our study confirmed the presence of tick Ago-1 and Ago-2 in the *I. scapularis *genome reads and found evidence for a complete Ago-1 protein in the *R. microplus *EST database. A *R. microplus *sequence containing a partial Ago-2 protein was also identified. The functions of these tick Argonaute proteins remains to be confirmed and further research is required to identify the full complement of tick PPD proteins.

Flies and mosquitoes do not possess *C. elegans *Sid-1 homologues known to be responsible for systemic and germ-line RNAi. Tick RNAi observed in this study and the literature demonstrate that a systemic RNAi silencing mechanism is active in ticks [42]. Although a tick Sid-1 was not found, we did however identify a tick homologue of the *C. elegans *EGO-1, an RNA dependent RNA polymerase (RdRP) known to amplify trigger dsRNA (transitive RNAi) and systemic RNAi [27,53]. RdRP is otherwise absent in flies, mosquitoes and other animals. Perhaps an RdRP-based RNAi amplification mechanism within the Ecdysozoans (including ticks and *C. elegans*) is common, but lost in insect species? Mechanisms for cell to cell dsRNA uptake within ticks requires further investigation, as well as the confirmation of the activity of the tick RdRP and Rsd-3 homologues identified here. Further research to identify putative tick secondary Argonautes associated with the transitive RNAi pathway in *C. elegans *is also warranted. These mechanisms have not been studied in spiders, mites or ticks to date, thus confirmation of RNAi mechanisms within the Cheliceromorpha will assist to confirm potentially new evolutionary mechanisms previously not defined and which cannot be based on pathways observed in insect species.

An additional aim of our study was to investigate whether fruit fly RNAi screens of conserved genes could be associated with similar tick phenotypes and tick gene function. We used a stringent search to enable the selection of the most similar sequences to maximize the probability of selecting a tick sequence which following dsRNA mediated knockdown could also affect growth and viability *in vitro*. With the exception of proteasome/ubiquitin protein homologues, the RNAi experiments with cultured *R. microplus *BME26 cells did not replicate the effects observed by Boutros and co-workers in *D. melanogaster *cells [43] for all targets. However, *in vivo *knockdown confirmed a lethal effect for 6 of the 10 targets, with only one demonstrating nil effects on tick reproduction. The *ubiquitin-63E *homologue which demonstrated the highest z score and impact on *Drosophila *cell viability exhibited the strongest effects on viability in our tick study both *in vitro *and *in vivo*. However the effects on tick cell growth and viability from the remaining 9 (including 2 negative controls) dsRNA targets tested did not correlate well with *Drosophila *demonstrating poor statistical significance at least under our *in vitro *conditions. Kurtti et al [54] found that cationic lipid-based reagents greatly improved the transfection of *I. scapularis *cultured tick cells as well as subsequent silencing of transgenes by dsRNAi. Perhaps uptake of nucleic acids by cultured tick cells is less efficient than with *Drosophila *cells. In addition, although tick genome resources are currently incomplete, we did not identify tick homologues of Scavenger receptors (Eater and SrCI) known to be required for dsRNA uptake in *Drosophila *cell culture [36]. This suggests the recruitment of different receptors for dsRNA uptake in tick cells compared to those described in *Drosophila*.

It is also possible that the cell line types utilized in *D. melanogaster *and *R. microplus *are not directly comparable and functionally different. The 438 genes targeted by Boutros and co-workers [43] compared the effects using 2 embryonic cell lines, Kc167 which is an 'early' embryonic cell line and S2R+ which is a 'late' embryonic cell line – potentially more comparable to BME26 cells which are 'late' embryonic in origin [55-57]. However, the BME26 average cell size is smaller at 15–20 μM compared to SR2+ cells at 50 μM [56,58]. The BME26 cell line also has a doubling time of 7 days, considerably slower than the *Drosophila *cell lines. Our *in vivo *studies were more convincing demonstrating lethal and inhibitory effects on tick reproduction for 9 (including the 2 controls) of the 10 targets. *In vivo *studies in *C. elegans *showed that 47% of the *C. elegans *orthologues of the 438 genes associated with the *Drosophila *RNAi phenotypes exhibited developmental phenotypes [43,59]. Perhaps the fact that we chose highly conserved homologues increased the probability of success in our *in vivo *experiments compared with *C. elegans*. However, it is clear that tick *in vitro *RNAi analysis using BME26 cannot be directly correlated to available *Drosophila in vitro *data, unless perhaps only target genes with higher z scores (as demonstrated here for *ubiquitin-63E*) can be studied to increase the probability of a phenotype correlation.

The challenges encountered during the initial PCR amplification of tick template DNA (results not shown) prompted re-design of conserved primer sets for targets amplified and transcribed in this study. Other tick dsRNA studies have used cDNA clones [42] as templates for amplification and subsequent transcription verifying that perhaps the tick genomic DNA templates are not amenable for high throughput gene amplification required for RNAi functional screens. The tick genome is large (7.1 Gbp) with a high ratio of repetitive and exonic sequences [2] also confirmed here with the *I. scapularis *putative Dicer genomic sequence structure with 14 exons. The presence of complex intronic/exonic structure can inhibit satisfactory PCR amplification of gDNA possibly due to poor primer binding. This was mostly overcome in this study by improving primers by targeting conserved ORFs across several arthropod species, however, amplification was not always consistent (not shown). It may be feasible to develop short interfering RNA treatments which would be simpler to prepare than long dsRNA treatments for difficult templates such as the tick, to date siRNAs have not been applied in *R. microplus *loss-off-function assays. Long dsRNA gene silencing can also lead to off target effects and false positive RNAi phenotypes [60,61]. Until complete annotated tick genome resources are available, false positive knockdown resulting from long dsRNA treatments and the specificity of (siRNAs) tick RNAi reagents cannot be confirmed.

## Conclusion

We utilized the existing *R. microplus *BmiGI2 database (13,643 ESTs) and the *I. scapularis *genome reads to identify 31 putative tick RNAi proteins which confirmed the presence of a putative Dicer, RISC associated, dsRNA uptake and RdRP proteins in ticks and constructed a putative tick RNAi pathway. Apart for proteasome/ubiquitinylation homologues, it was not feasible to replicate *D. melanogaster *embryonic cell culture RNAi functional data in *R. microplus *BME26 embryonic cells. This could either be attributed to transfection/uptake issues and/or a difference in cell types in the fly and tick embryonic cell lines. We did demonstrate a correlating *in vivo *effect on embryogenesis for 9 of the 10 *D. melanogaster *tick homologues. The findings in this manuscript support the fact that perhaps the Chelicerates may not be amenable to modeling based on insect pathways (Subphylum Mandibulata) as perhaps expected for Arthropods. With the evidence of a tick RdRP and the propensity for systemic or germ-line RNAi, it will be better to compare gene function and RNAi pathways between members of the Arachnida and the Superphylum Nemathelminthes (*C. elegans*). Until more tick and related genomes (mites and spiders) are available, such comparative studies within these Subphyla are not feasible. Clearly the RNAi pathways warrant further elucidation, and tick specific genome and functional data will be beneficial for tick research and for the development of improved tick control measures.

## Methods

### Sources of input sequence data

13,643 ESTs (9,403 Tentative Consensus/TC and 4,240 singleton) sequences for *R. microplus *were obtained from the *Boophilus microplus *Gene Index (BmiGI) at  (last accessed: 17/6/2008)[3]. *I. scapularis *(black legged tick) genome project (IGP) data was accessed through  (last accessed: 13/6/2008); and 38,276 *I. scapularis *EST sequences were obtained from the *Ixodes scapularis *Gene Index (ISGI) at  (last accessed: 17/6/2008). All other nucleotide and amino acid sequences were obtained from the Entrez nucleotide and protein databases .

### Identification of conserved genes in *R. microplus*

Key RNAi pathway-associated proteins from *D. melanogaster *and *C. elegans *described previously [28,32,35,36,45,46,50,73,74] were screened against the available tick ESTs (BmiGI2) [3] and *I. scapularis *genome contig reads obtained from the NCBI whole genome project database (project ID 16233) using BLAST [62]. For the BLAST searches an initial e-value of <1e-05 was set as a threshold. The best hits from the *R. microplus *and *I. scapularis *sequences where then used as query sequences in a second round of BLAST searches against the *D. melanogaster *and *C. elegans *subsets of the NCBI non-redundant protein database. Results of this reciprocal BLAST search validated the sequence similarity between the key RNAi pathway-associated proteins from *D. melanogaster *and *C. elegans *and the two ixodid tick species, sequences which did not return the corresponding RNAi protein were subsequently disregarded. Further confirmation was obtained by performing searches against the InterPro database using InterProScan (data not shown) [63]. All searches were performed with the BLAST default settings. Specific approaches for Dicer, Argonautes and RdRP homologues are described below.

#### Dicer domains

Multiple sequence alignments for the domains typical for proteins of the Dicer family were retrieved from the Pfam website. Specifically these were the alignments for the Helicase conserved C terminal domain (Pfam:PF00271), double-stranded RNA binding domain (Pfam:PF03368), PAZ domain (Pfam:PF02170), RNase3 domain (Pfam:PF00636), and the double-stranded RNA binding motif (Pfam:PF00035). Hidden Markov Models (HMMs) were constructed locally using hmmbuild of the HMMER2 package [64] with default settings. The programs estwisedb and genewisedb of the Wise2 package [65] were used to perform searches with each HMM as a query in a local copy of the BmiGI2 database and the *I. scapularis *sequences obtained from NCBI whole genome sequencing projects. The best hit sequences from these searches were retrieved from the respective databases and a conceptual translation of encoded open reading frames (ORFs) was performed using the program getorf, part of the EMBOSS package of computational biology tools. The ORFs were then used as the query sequence for a blastp search against the NCBI Reference Sequence protein database to verify the validity of the initial search results. Further confirmation of the search results was achieved by screening the ORFs against the Pfam database using the global search model.

For the prediction of gene models and the identification of the exon/intron structure, the program genewise from the Wise2 package was used to map the detected ORFs to the genomic sequences. *I. scapularis *expressed sequence tags from the *I. scapularis *ISGI2 database were used in blastn searches to verify the validity of the predicted exon/intron structure by genewise. The sequences of the ORFs were also screened against a local copy of the Pfam database using the program hmmpfam of the HMMER2 package to reveal the sequence structure of the conserved domains.

#### Argonaute domains

Multiple sequence alignments of amino acid sequences stored in the Pfam database were obtained for following protein domains and domain families: Domain of unknown function (DUF)1785 (Pfam:PF08699), PAZ domain (Pfam:PF02170) and Piwi (Pfam:PF02171). HMMs were built from the multiple sequence alignments using the program hmmbuild with default settings. The BmiGI2 database and *I. scapularis *sequences were searched with the programs estwisedb and genewisedb using the HMMs as query sequences. The program getorf was used to conceptually translate the ORFs of the best hits. The validity of the initial search results was verified by blastp searches against the NCBI Reference Sequence protein database. Further confirmation of the search results was achieved by screening the ORFs against the Pfam database using the global search model. A comparison between known Argonaute proteins from *D. melanogaster *and *C. elegans *was performed using the program bl2seq, which uses the BLAST algorithm for a pairwise comparison.

#### Phylogenetic analysis of Dicer, Argonaute and RNA-dependent RNA polymerase proteins

Multiple sequence alignments of the protein sequences and ORFs were performed using Clustalw [66] with default program settings. In addition to the Dicer sequences illustrated in Figure 1a, following protein sequences were included in the construction of the phylogenetic tree (Figure 1b): *Bos taurus *Dicer-1 [GenBank:NP_976235.1] and *T. castaneum *Dicer-2 [GenBank:NP_001107840.1]. The phylogenetic trees (Figures 2b and 3b) for Argonaute-1 and Argonaute-2 were constructed using additional sequences from *C. elegans *[GenBank:NP_510322.2] and [GenBank:NP_871992.1], *T. castaneum *[GenBank:XP_971295.2] and [GenBank: NP_001107842.1] and *B. taurus *[GenBank:NP_991363.1] and [GenBank:AAS21301.1]. For both Argonaute-1 and 2 proteins, the phylogenetic trees were based on the alignments of the DUF1785 and PAZ domains. The phylogenetic tree (Figure 4) for the partial *R. microplus *RdRP protein (Cat-RdRP) was constructed using additional RdRP domain (Pfam:05183) sequences from the metazoans: *C. elegans *(Ego-1 [GenBank:NP_492132.1], rrf-1 [GenBank:NP_492131.1] and rrf-3 [GenBank:NP_495713.1]) and *I. scapularis *([GenBank:EEC04985.1], [GenBank:EEC05952.1], [GenBank:EEC12509.1], [GenBank:EEC12909.1]); plants: *Arabidopsis thaliana *[GenBank: NP_172932], *Hordeum vulgare *[GenBank:ACH53360.1], *Nicotiana tabacum *[GenBank:CAR47810.1], and *Solanum lycopersicum *[GenBank:ABI34311.1]; fungi: *Aspergillus fumigatus *[GenBank:EDP48577.1], *Neurospora crassa *[GenBank:XP_964248.2] and *Schizosaccharomyces pombe *[GenBank:NP_593295.1]; and protists: *Dictyostelium discoideum *[GenBank:XP_636093.1] and *Tetrahymena thermophila *[GenBank:XP_001026321.1]. The conserved RdRP domains were extracted from these sequences and then used for the alignment to the partial *R. microplus *RdRP domain. The multiple sequence alignments for all 3 studied proteins were visually inspected and the phylogenetic trees were constructed using Geneious 3.8.5 , last accessed on 12/08/08). Pairwise distances were calculated based on the BLOSUM62 matrix and the respective trees were constructed using Neighbor-Joining. No outgroups were selected and the consensus trees were built using bootstrapping with 5,000 samples.

### Identification of *R. microplus *homologues for known *Drosophila *RNAi viability phenotypes

Raw experimental results of the genome wide RNAi screen of *D. melanogaster *are publicly available at the website [67]. The gene ontology data for the identified RNAi targets were retrieved from [4]. Genes of interest were selected based upon a phenotypic z score > 3 [43]. For these genes, corresponding translations were retrieved from FlyBase and used for the subsequent amino acid similarity searches. The *D. melanogaster *cDNA sequences from the selected RNAi targets were used to screen to search the 13,643 of the *R. microplus *ESTs and TCs for highly conserved genes using blastn [62]. Sequences with a similarity of at least 80% and an e-value less than e1^-50 ^were selected, conceptually translated and their putative function was further analyzed by assigning GO terms using InterProScan [63,68,69]. Additional Files 2 and 3 describe this selection process and the GO terms utilized respectively.

### Tick cell culture and sources of ticks for dsRNA treatment studies

BME26 was derived in 1985 from *R. microplus *embryonated eggs in the USA [57] and supplied by Dr. Munderloh (Department of Entomology, University of Minnesota, St. Paul, Minnesota 55108) to the Queensland Department of Primary Industries & Fisheries in Australia. Cell culture protocols to maintain and passage the cell line (obtained at passage 55) have been previously described [70]. N strain adult female ticks were obtained from the DPI&F Animal Research Institute tick cell colony [71].

### DNA and RNA extraction methods

DNA from BME26 cells was prepared using the QIAamp DNA mini kit (QIAGEN Sciences, MD, USA) – protocol for cultured cells as described by the manufacturer. RNA for qRT-PCR analysis prepared from BME26 cells, adult tick viscera, and tick eggs was extracted using TRIzol^® ^reagent (Invitrogen, CA, USA) following the manufacturer's instructions. For RNA extractions from larvae, the larvae were first ground in liquid nitrogen using a mortar and pestle prior to TRIzol^® ^reagent extraction following the manufacturer's instruction (Invitrogen, CA, USA).

### dsRNA synthesis methods

Sequences from *Anopheles gambiae, D. melanogaster, I. scapularis*, and *R. microplus *(Additional File 4) were aligned using AlignX (Invitrogen Vector NTI, CA, USA) to identify conserved regions for primer design. Primers were subsequently designed using Invitrogen Vector NTI to amplify the corresponding conserved region in *R. microplus *(Additional File 5). T7 promoter sequences were added to the 5'-ends of the primers to allow for subsequent RNA transcription as described in the manufacturer's instructions (Ambion MEGAScript RNAi kit, Applied Biosystems, CA, USA). PCR products were amplified from 20 ng DNA prepared from BME26 cells as template using 10 pM each primer, 10 pmol dNTPs, HotStart *Taq *Plus enzyme and the buffer provided by the manufacturer (QIAGEN Sciences, MD, USA) in a 20 μl reaction volume. The optimal annealing temperature for each assay was determined using gradient PCR and a temperature gradient of 55°C to 70°C in twelve discrete steps in a G-storm GS-1 thermocycler (Geneworks Technologies Pty Ltd, SA, Australia). The PCR thermal profile was as follows: 95°C 2 min, followed by 35 cycles at 95°C 10s, annealing temp 30s, 72°C 1 min (annealing temperatures for each primer pair described in Additional File 5), and a final extension at 72°C 7 min. The size of the PCR products (Additional File 5) were confirmed by gel electrophoresis using 1.5% Agarose in TAE Buffer (Tris acetate 40 mM, EDTA 2 mM, pH 8.5) after 45 minutes at 90 V. The PCR products were purified using the QIAquick kit (QIAGEN Sciences, MD, USA) following the manufacturer's protocol. Long dsRNA were synthesized from the purified PCR products (5 pooled 20 μl reactions per gene target) using the MEGAScript RNAi kit as described by the manufacturer (Ambion, Applied Biosystems, CA, USA). Purified dsRNAs were stored in elution buffer at -70°C until further use. Actin (TC12168) and the dsRNA control supplied by the manufacturer (MEGAScript, Ambion, Applied Biosystems, CA, USA) were prepared as tick specific and non-specific dsRNA treatments, respectively.

### Transfection of BME26 tick cells

*In vitro *transfection methods for the dsRNA treatment of tick cells were modified from *D. melanogaster *methods originally described by Boutros et al [43]. BME26 cells at passage 57 were grown in 96-well plates freshly seeded with 48,000 cells/40 μl per well. Cells were transfected with 800 ng dsRNA and incubated at 31°C for 60 mins prior to the addition of complete medium (final total well volume of 120 μl). Treatments were incubated for 4 days at 31°C and each well was supplemented with 80 μl complete medium at Day 2. Each treatment contained 6 replicates to provide 3 replicates for viability assay and 3 for qRT-PCR. On Day 4 (96 hrs post treatment), 3 wells were subjected to cell viability testing using the Cell Glow kit as per manufacturer's instructions (Promega Corporation, WI, USA) and 3 wells were subject to RNA extraction for qRT-PCR screening. Controls included nil treatment (media only) and the dsRNA control from the Ambion MEGAScript RNAi kit (non-specific dsRNA treatment). Impairment of growth and viability relative to the nil treatment control was statistically determined by calculating inverse z-scores for every treatment [43].

### Injection of *R. microplus *ticks with dsRNA, monitoring and statistical analysis of mortality and egg output

Female adult ticks fed to repletion were collected within 24 hrs from dropping from the bovine host for dsRNA injection. Six ticks per treatment (10 *Drosophila *homologues, no injection control, PBS injection control, tick actin dsRNA and the MEGAScript dsRNA control) were injected with 1–2 × 10^12 ^dsRNA molecules using a micro-injector (World Precision Instruments Inc., Florida, USA) as described previously by Nijhof and colleagues [42], except ticks were first pierced using a 30 G needle rather than 27 G. Five out of the 6 ticks per treatment were monitored daily for effects on mortality, egg output and larval hatching rates until all ticks had died [42]. Statistical analyses were conducted using GenStat 10 (VSN International). The following variables were subjected to analysis of variance assessing the effect of replicates and treatments: 1. total wt of eggs produced; 2. days ticks survived post injection; 3. days from laying to larval hatch; and 4. percent larvae hatched. A protected least significant difference (LSD) procedure was used to compare treatment means using a significance level of 0.05. RNA was extracted from the viscera and from the eggs collected from the 6^th ^replicate tick per treatment for qRT-PCR analysis on days 6 and 14 respectively (see below).

### Quantitative RT-PCR gene expression analysis

Primer sequences, PCR product and annealing temperatures for all targets are described in Additional File 5. cDNA was synthesized using a cDNA synthesis kit (Bioline International, London, UK), and triplicate qPCRs (50 ng per reaction) of BME26 cells was undertaken using SensiMixPlus SYBR kit (Quantace Ltd, Watford, UK) in the Corbett RotorGene 3000 (Corbett, Sydney, Australia) using the following profile: 95°C 10 mins; 40 cycles of 95°C 15 s, 55°C 30 s, 72°C 30 sec, followed by a melt analysis 72–90°C 30 s on the first step, 5 s holds for subsequent steps, according to manufacturer's instructions for SYBR green detection. All the results corresponded to relative quantification using *R. microplus *actin (Additional File 5) as an internal control gene using the 2^-ΔΔCt ^method [72].

Viscera from the 6^th ^replicate tick of each *Drosophila *homologue group were homogenized in TRIzol^® ^to extract total RNA. The semi quantitative analysis of the samples was undertaken using the QuantiTect SYBR green RT-PCR Kit^® ^(QIAGEN, Australia) as recommended by the manufacturer. The expression profiles were normalised against *R. microplus *actin as above. Reactions contained 125 ng of total RNA, 12.5 μl of 2× QuantiTect SYBR Green RT-PCR Master mix, 10 pmol of each primer, 0.25 μl QuantiTect RT Mix, the final reaction volume was 25 μl. RT-PCR reaction were conducted on Rotor-Gene 3000 under the following conditions: reverse transcription 50°C for 30 min, PCR initial activation at 95°C for 15 min, followed by 40 cycles at 94°C, 15 s, 55°C, 30 s and 72°C 30 s. Calculation of percent gene expression and knockdown (average of 3 triplicate reactions) was determined by comparative C_T _method for relative quantification as described above.

### *R. microplus* EST Accessions

GenBank Accessions describing the *R. microplus* ESTs identified in this study have been appended as Additional File 6.

## Abbreviations

dsRNA: double-stranded RNA; cDNA: complementary DNA; EST: expressed sequence tags; GO: Gene Ontology; PCR: polymerase chain reaction; RISC: RNA-induced silencing complex; RNAi: RNA interference; siRNA: small interfering RNA;

## Authors' contributions

SK conducted the bioinformatics analysis and the *in vitro *transcription of dsRNA and is one of the senior authors of this manuscript.

AL directed most laboratory activities and provided contextual details in regard to bioinformatics searches and RNAi pathways. AL contributed equally with SK in the preparation of this manuscript.

MRV conducted the qRT-PCR experiments and authored the corresponding sections.

AB conducted the dsRNA injection experiments and authored the corresponding results and methods sections.

VD undertook statistical analyses and interpretation of results.

UM provided the BME26 cell line and authored descriptions within the manuscript thereof.

FG provided the BmiGI ESTs, assisted with project design and manuscript edits.

MB and RB directed the bioinformatics analyses with considerable input into direction of the research.

## Supplementary Material

Additional File 1 **Table of conserved domains of Dicer proteins identified in *R. microplus *ESTs with details on ORF length, domain positions, result scores of Pfam search, and scores of BLAST searches.** List of Dicer domains identified ESTs in *R. microplus*. Click here for file

Additional File 2 **Bioinformatics analysis pipeline.** A dataflow diagram of the bioinformatics analysis pipeline used in the identification of RNAi targets. Click here for file

Additional File 3 **Gene Ontology terms distribution of *R. microplus *sequences.** A schematic representation of the functional relationship of the *R. microplus *genes targeted in the RNAi cell culture and *in vivo *experiments, based on Gene Ontology terms assigned by InterProScan searches. Click here for file

Additional File 4 **Sequences used in the identification of conserved regions for the design of primers for the PCR amplification of *R. microplus *homologues of *D. melanogaster *known RNAi phenotypes.** List of GenBank accessions used to identify conserved regions to assist with primer design for the amplification of dsRNA treatments. Click here for file

Additional File 5 **Sequences of oligonucleotides used for the amplification of template DNA for subsequent *in vitro *transcription of dsRNA.** Click here for file

Additional File 6 **GenBank accessions for clones for *R. microplus *tentative consensus sequences identified in this study.** Click here for file
