# Supplementary material for: Evidence of a tick RNAi pathway by comparative genomics and reverse genetics screen of targets with known loss-of-function phenotypes in Drosophila
Source: BMC Mol Biol. 2009 Mar 26;10:26. doi: 10.1186/1471-2199-10-26 (PMC2676286; doi:10.1186/1471-2199-10-26)
Supplement: Additional File 2 — Bioinformatics analysis pipeline. A dataflow diagram of the bioinformatics analysis pipeline used in the identification of RNAi targets. [file 1471-2199-10-26-S2.ppt]

## Slide 1
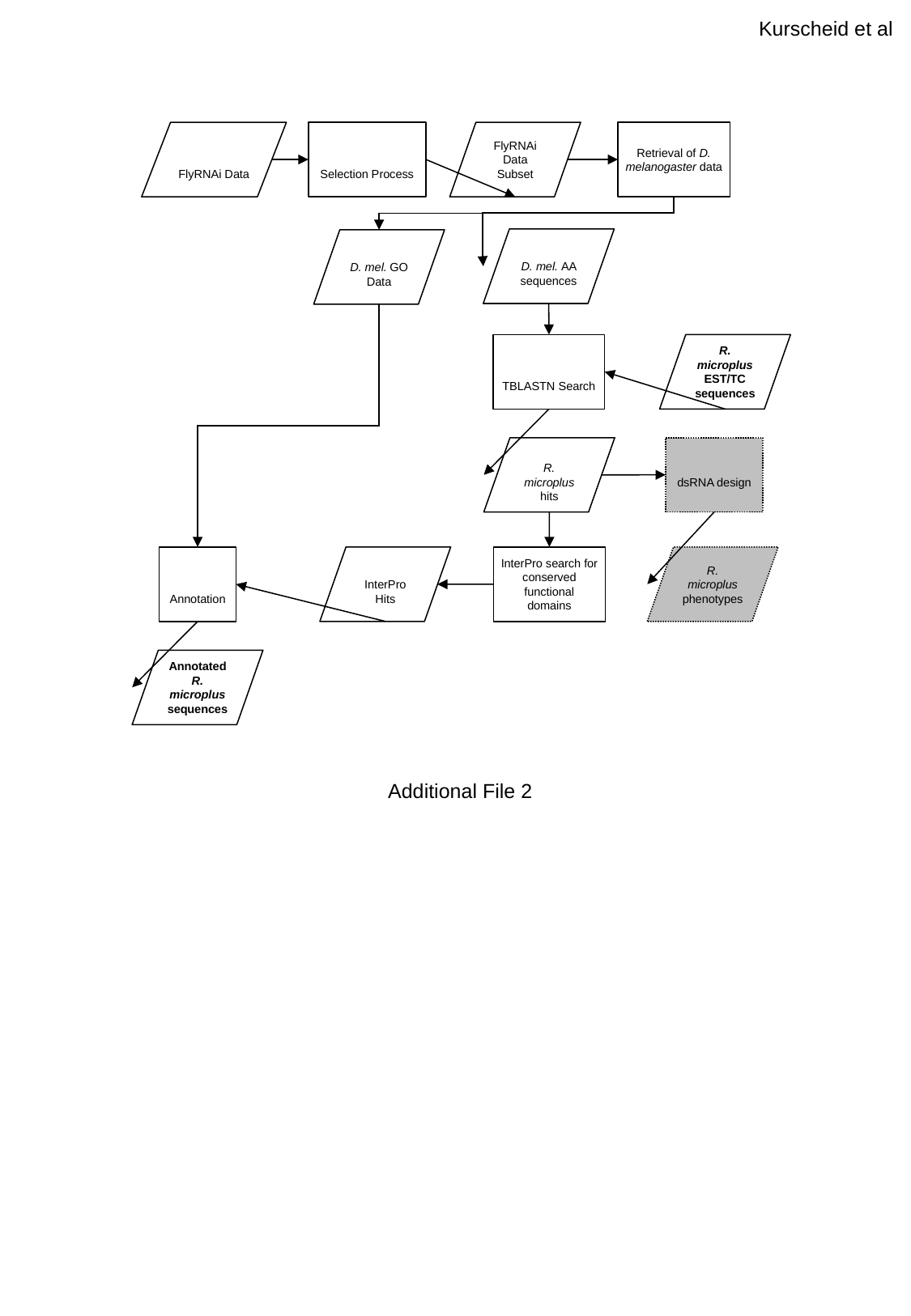

Kurscheid et al
FlyRNAi Data
Selection Process
FlyRNAi Data Subset
Retrieval of D. melanogaster data
D. mel. AA sequences
D. mel. GO Data
TBLASTN Search
R. microplus EST/TC sequences
R. microplus hits
dsRNA design
Annotation
InterPro Hits
InterPro search for conserved functional domains
R. microplus phenotypes
Annotated R. microplus sequences
Additional File 2
