# Supplementary figures and images for: Evidence of a tick RNAi pathway by comparative genomics and reverse genetics screen of targets with known loss-of-function phenotypes in Drosophila
Source: BMC Mol Biol. 2009 Mar 26;10:26. doi: 10.1186/1471-2199-10-26 (PMC2676286; doi:10.1186/1471-2199-10-26)

## Slide 1
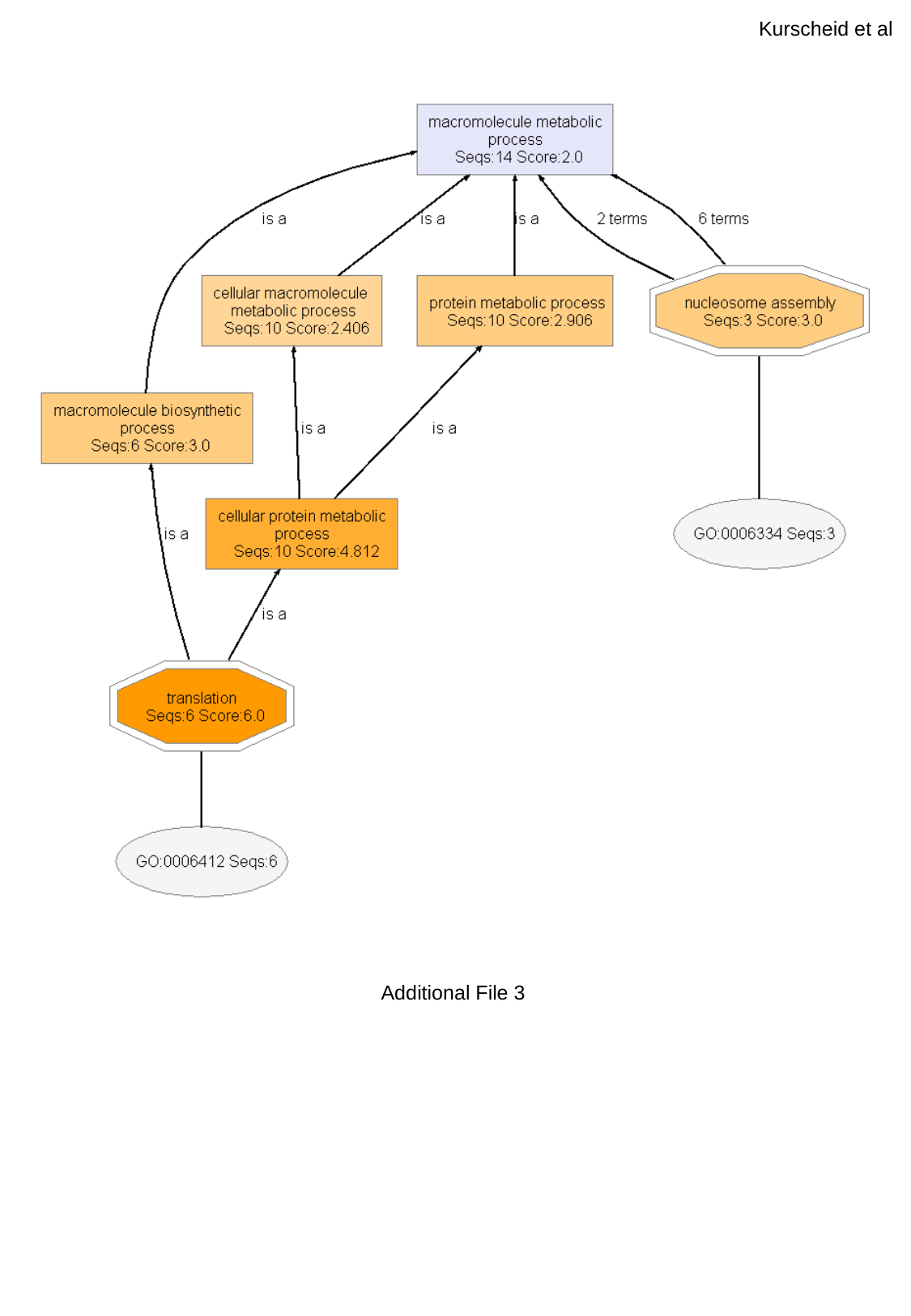

Kurscheid et al
Additional File 3

Supplement: Additional File 3 — Gene Ontology terms distribution of R. microplus sequences. A schematic representation of the functional relationship of the R. microplus genes targeted in the RNAi cell culture and in vivo experiments, based on Gene Ontology terms assigned by InterProScan searches. [file 1471-2199-10-26-S3.ppt]
