# Supplementary material for: Evidence of a tick RNAi pathway by comparative genomics and reverse genetics screen of targets with known loss-of-function phenotypes in Drosophila
Source: BMC Mol Biol. 2009 Mar 26;10:26. doi: 10.1186/1471-2199-10-26 (PMC2676286; doi:10.1186/1471-2199-10-26)
Supplement: Additional File 4 — Sequences used in the identification of conserved regions for the design of primers for the PCR amplification of R. microplus homologues of D. melanogaster known RNAi phenotypes. List of GenBank accessions used to identify conserved regions to assist with primer design for the amplification of dsRNA treatments. [file 1471-2199-10-26-S4.doc]

**Additional File 4. Sequences used in the identification of conserved regions for the design of primers for the PCR amplification of *R. microplus* homologues of *D. melanogaster* known RNAi phenotypes.**

| ***R. microplus* BmiGI2 ID** | ***A. gambiae* GenBank Accession** | ***D. melanogaster* GenBank Accession** | ***I. scapularis* sequence ID (ISGI)** |
| --- | --- | --- | --- |
| TC5762 | DR747973.1 | NM_057526.3 | G894P562FJ13.T0 |
| TC5823 | BX610015.1 | NM_166808.1 | G893P554RM22.T0 |
| TC6116 | XM_320335.2 | NM_078755.2 | G894P557RG6.T0 |
| TC9037 | XM_309477.3 | NM_057706.3 | G894P553RC20.T0 |
| TC9417 | XM_310958.3 | NM_134690.2 | G893P559RJ17.T0 |
| TC9852 | XM_312924.3 | AY058759.1 | G894P539RP14.T0 |
| TC12182 | AAAB01067499.1 | NM_164624.1 | 1099712033590 |
| TC12306 | XM_315817.3 | NM_167955.1 | G893P540RG5.T0 |
| TC12372 | BX069580.1 | NM_140257.1 | G894P562FJ13.T0 |
| TC12393 | BX023300.1 | NM_057590.3 | G894P528RB6.T0 |
| TC13930 | XM_310465 | NM_079740 | G894P541RK16.T0 |
| Actin (TC12168) | U02930.1 | NM_169525.1 | G894P552RB24.T0 |
