# Supplementary material for: Evidence of a tick RNAi pathway by comparative genomics and reverse genetics screen of targets with known loss-of-function phenotypes in Drosophila
Source: BMC Mol Biol. 2009 Mar 26;10:26. doi: 10.1186/1471-2199-10-26 (PMC2676286; doi:10.1186/1471-2199-10-26)
Supplement: Additional File 5 — Sequences of oligonucleotides used for the amplification of template DNA for subsequent in vitro transcription of dsRNA. [file 1471-2199-10-26-S5.doc]

**Additional File 5. Sequences of oligonucleotides used for the amplification of template DNA for subsequent *in vitro* transcription of dsRNA.**

| ***R. microplus* BmiGI2 ID** | **^1^Primer sequences 5’ – 3’** | **Annealing Temp [^o^C]** | **product size [bp]** |
| --- | --- | --- | --- |
| TC5762 | Rmi.TC5762cF AACAGTGCGGGCAGTGCCGA  Rmi.TC5762cR  CTCGGCGTCAAGTGCTCCAAGG | 58 | 444 |
| TC5823 | Rmi.TC5823F CATTCCCTCGGACAAGTTCC  Rmi.TC5823R  TTTGGTGGTGGTAATACGTTCC | 59 | 580 |
| TC6116 | Rmi.TC61661F TGAAATTAAGTGCGCAGAAC  Rmi.TC6116R CTCTTCTACACGCATCATCG | 60 | 390 |
| TC6372 | BmTC6372F  CGCACTTTGTCCGACTACAA  BmTC6372R AGGCTGGTCAACACAAAACC | 60 | 600 |
| TC9037 | Rmi.TC9037F TGTGAACAGCAGGTCTATTTGC  Rmi.TC9037R TTCTCCAAGGCTCGCTACAC | 60 | 413 |
| TC9417 | Rmi.TC9417F AGACATGCCAGGAGGATAAGC  Rmi.TC9417R ACACGTTTCTGCCACAATGG | 55 | 482 |
| TC9852 | Rmi.TC9852cF GTGCTCGCTACCCACGGATGTT  Rmi.TC9852cR CAAACACGAACCCCCGATTCC | 65 | 325 |
| TC10417 | BmTC10417F  ATCTCCTTGGTGTCGTTTGC  BmTC10417R AATGCCACAGCTCTCTTGCT | 60 | 600 |
| TC12182 | Rmi.TC12182F  TGGCTCGCACCAAGCAGACAGC  Rmi.TC12182R CGCTCGTTCTCCGCGGATGC | 60 | 407 |
| TC12306 | Rmi.TC12306cF TCCAGGTGGGCAATGTGCTTCC  Rmi.TC12306cR CTTGCGACCAGCAGAGGCATCC | 60 | 416 |
| TC12372 | Rmi.TC12372F CTATACGACTGCGTCAATGC  Rmi.TC12372R GGACACCAGGTAGTTGATGG | 60 | 549 |
| TC12393 | Rmi.TC12393F GTATGCACTGTAGTGGCAAGG  Rmi.TC12393R TACGCTTTGCCTTGTAGTAGC | 60 | 393 |
| TC13930 | Rmi.TC13930F CCCAGAAAGAGAAGATCAAGG  Rmi.TC13930R CCCTTGGGAGGACTTATACC | 60 | 466 |
| TC12168 (Actin) | Rmi.ActinbF CACGCCCTTCGCCGGTGTTCG  Rmi.ActinbR ACCGCCGCCATCTTGCGAAAGC | 60 | 513 |

^1^T7 sequences (TAATACGACTCACTATAGGG) added to 5’ end of oligonucleotide sequence for dsRNA amplification and transcription as described in manufacturer’s protocol (MEGAScript, Ambion). The primer sets without T7 adaptor were used in qRT-PCR to confirm knockdown efficienc
